# Personalized Computational Modeling of Mitral Valve Prolapse: Virtual Leaflet Resection

**DOI:** 10.1371/journal.pone.0130906

**Published:** 2015-06-23

**Authors:** Yonghoon Rim, Ahnryul Choi, David D. McPherson, Hyunggun Kim

**Affiliations:** Division of Cardiovascular Medicine, Department of Internal Medicine, The University of Texas Health Science Center at Houston, Houston, Texas, United States of America; Sapienza University of Rome, ITALY

## Abstract

Posterior leaflet prolapse following chordal elongation or rupture is one of the primary valvular diseases in patients with degenerative mitral valves (MVs). Quadrangular resection followed by ring annuloplasty is a reliable and reproducible surgical repair technique for treatment of posterior leaflet prolapse. Virtual MV repair simulation of leaflet resection in association with patient-specific 3D echocardiographic data can provide quantitative biomechanical and physiologic characteristics of pre- and post-resection MV function. We have developed a solid personalized computational simulation protocol to perform virtual MV repair using standard clinical guidelines of posterior leaflet resection with annuloplasty ring implantation. A virtual MV model was created using 3D echocardiographic data of a patient with posterior chordal rupture and severe mitral regurgitation. A quadrangle-shaped leaflet portion in the prolapsed posterior leaflet was removed, and virtual plication and suturing were performed. An annuloplasty ring of proper size was reconstructed and virtual ring annuloplasty was performed by superimposing the ring and the mitral annulus. Following the quadrangular resection and ring annuloplasty simulations, patient-specific annular motion and physiologic transvalvular pressure gradient were implemented and dynamic finite element simulation of MV function was performed. The pre-resection MV demonstrated a substantial lack of leaflet coaptation which directly correlated with the severe mitral regurgitation. Excessive stress concentration was found along the free marginal edge of the posterior leaflet involving the chordal rupture. Following the virtual resection and ring annuloplasty, the severity of the posterior leaflet prolapse markedly decreased. Excessive stress concentration disappeared over both anterior and posterior leaflets, and complete leaflet coaptation was effectively restored. This novel personalized virtual MV repair strategy has great potential to help with preoperative selection of the patient-specific optimal MV repair techniques, allow innovative surgical planning to expect improved efficacy of MV repair with more predictable outcomes, and ultimately provide more effective medical care for the patient.

## Introduction

Posterior leaflet prolapse following chordal elongation or rupture is one of the primary valvular diseases in patients with degenerative mitral valves (MVs) [1]. Leaflet prolapse is commonly accompanied with varying degrees of mitral regurgitation (MR) due to leaflet malcoaptation during systole [1, 2]. When one or more posterior marginal chordae are ruptured, posterior leaflet prolapse immediately occurs leading to distension of the leaflets and increase in the tensional stress in the remaining chordae under high pressure loading [3]. Distended posterior leaflet tissue and ruptured chordae need to be repaired to restore normal geometry and MV function and prevent further pathologic progression [3–5]. MV repair is well-known to be generally superior to total MV replacement in patients who have degenerative MV diseases [6]. Clinical long-term outcomes demonstrate that quadrangular resection followed by annular plication and/or sliding leaflet plasty is a reliable and reproducible surgical repair technique for treatment of posterior leaflet prolapse [1, 6, 7].

Although resection is considered one of the best interventional choices for treatment of posterior leaflet prolapse, prosthetic valve replacement is often performed rather than MV repair. In many cases, this sub-optimal selection of surgical treatment is attributed to the surgeon’s discomfort with the complicated repair techniques that require extensive surgical experience [8]. Virtual MV repair simulation using patient-specific MV geometric data can be a powerful tool to provide computer-predicted surgical outcomes following resection repair and help better prepare for planning of detailed surgical procedures [9].

Computational techniques including finite element (FE) analysis have been actively utilized to provide valuable additive information to evaluation of heart valve diseases, particularly from biomechanical perspectives [10–15]. In recent studies, computational simulations of MV function combined with patient-specific three-dimensional (3D) echocardiography, magnetic resonance imaging (MRI) or computed tomography (CT) demonstrate the great potential for improved diagnosis of MV pathophysiology and advanced surgical planning of interventional treatment [16–22]. Virtual MV repair simulation of leaflet resection in association with patient-specific 3D echocardiographic data can provide quantitative information pertaining to the biomechanical and physiologic characteristics of pre- and post-resection MV function.

In the present study, a novel personalized computational simulation protocol for virtual surgery for posterior leaflet resection is described. A patient MV with ruptured posterior chordae tendineae was virtually repaired following the standard surgical techniques for leaflet resection and ring annuloplasty. Dynamic simulations of MV function before and after virtual resection were performed, and the effects of the repair on restoration of normal MV function were investigated.

## Materials and Methods

### Personalized Computational MV Modeling

**Fig 1:**
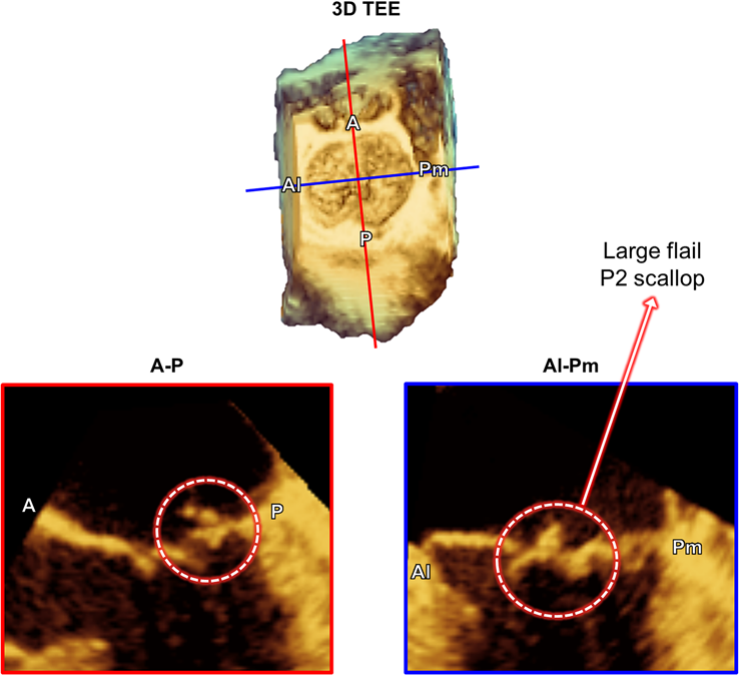
MV geometric information from the 3D TEE data of a patient with ruptured posterior chordae tendineae. (top) Volumetric en-face view of the MV, and (bottom) cross-sectional images of the annulus and leaflets in the antero-posterior (A-P) and anterolateral-posteromedial (Al-Pm) planes.

This translational study to utilize patient 3D transesophageal echocardiographic (TEE) data was approved by the Committee for the Protection of Human Subjects at The University of Texas Health Science Center at Houston. Written informed consent forms were collected from the participants. We have recently developed a novel personalized computational protocol to perform virtual MV modeling and repair simulation using MATLAB (The Mathworks Inc., Natick, MA, USA) and ABAQUS (SIMULIA, Providence, RI, USA) software [18, 23]. Briefly, 3D geometric information of the MV apparatus (the annulus, the anterior and posterior leaflets, and location of the papillary muscles) was retrospectively extracted from 3D TEE data (iE33 Ultrasound System, Philips Healthcare, Andover, MA, USA) of a patient with ruptured posterior chordae tendineae. The leaflets and annulus were segmented, modeled using the 3D non-uniform rational B-spline (NURBS) surface modeling technique, and meshed. This particular patient had severe posterior leaflet prolapse with a large flail due to chordal rupture of the P2 scallop (Fig 1). As the actual chordal distribution information was not available from 3D TEE data, an average number of the marginal and strut chordae [24] were added to the leaflets. Approximately 70% of the chordae in the P2 scallop region were removed to create P2 chordal rupture and prolapse. The chordae insertion was distributed around the papillary muscle tips that were located from the patient 3D TEE data.

### Physical Properties of the MV Leaflets and Chordae Tendineae

The stress-strain relationship was implemented into the circumferential (σ_*c*_) and radial (σ_*r*_) directions by fitting the biaxial mechanical testing data of the anterior and posterior leaflet tissue from a previous study [25]. The Fung-type hyperelastic material model was implemented into ABAQUS/Explicit to account for the characteristics of large deformation of the leaflets [17, 23]. Leaflet thicknesses of the anterior and posterior leaflets were set to be 0.69 mm and 0.51 mm, respectively [26]. The chordae tendineae were modeled as nonlinear hyperelastic materials using the Ogden models for the marginal and strut chordae [13]. Cross-sectional areas of the chordae tendineae were set to be 0.29 mm^2^ for the anterior marginal chordae, 0.27 mm^2^ for the posterior marginal chordae, and 0.61 mm^2^ for the strut chordae, respectively [13]. Physiologic density (1,100 kg/m^3^) and Poisson’s ratio (0.48) were appropriately employed for the whole MV apparatus tissue [10, 11, 15].

### Virtual Leaflet Resection and Ring Annuloplasty

**Fig 2:**
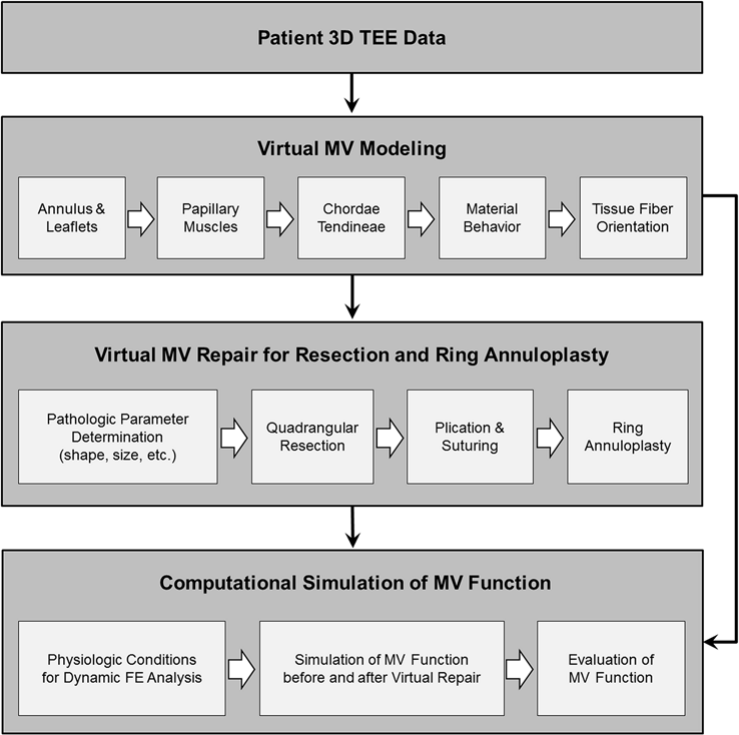
Personalized computational modeling protocol for virtual MV repair to perform leaflet resection and annuloplasty ring implantation followed by dynamic simulation of MV function.

Fig 2 demonstrates our computational simulation protocol for virtual MV repair to perform leaflet resection and annuloplasty ring implantation. Following virtual modeling of the patient MV from 3D TEE data, primary pathologic parameters of the MV were determined to plan for virtual repair simulation. All virtual MV repair protocols were designed following the standard clinical guidelines of MV repair surgery [3, 27]. As the patient had a large flail in the P2 scallop due to chordal rupture, a quadrangular leaflet resection was virtually performed in the P2 region followed by ring annuloplasty.

A quadrangle-shaped leaflet portion in the prolapsed P2 scallop was defined for resection such that two excision curves (~15 mm) connecting from the annulus to the leaflet free margin were delineated 5 mm apart from the remaining intact native marginal chordae in the P2 region. Once the elements in this pre-defined resection region were removed, a predicted virtual 3D curvature configuration for virtual plication and suturing was determined such that the key points of the curvature was located at the same distance from the pairwise nodes along the two excised edges of the posterior leaflet, and a cubic spline interpolation method was used to create a smooth 3D curvature configuration. Virtual annular plication was performed by deforming the two excised leaflet edges to the suturing configuration via imposing appropriate nodal displacements. These two excised leaflet edges were combined to complete virtual suturing. During this virtual annular plication and suturing procedure, the anterior annulus was fixed to maintain the comparable annular size and shape while the posterior annulus was deformed.

Improper sizing of the base of the anterior leaflet followed by inadequate ring selection often causes unsatisfactory repair outcomes [3]. The standard clinical guidelines of ring annuloplasty suggest selection of proper ring size based on the measurement of the width and height of the anterior leaflet. Following the measurement of the dimension of the anterior leaflet and the mitral annulus, the geometric profile of an annuloplasty ring (Physio II, Edwards Lifesciences, Irvine, CA, USA) of proper size (34 mm) was reconstructed using a 3D cubic spline algorithm [28]. A size 34 mm ring was chosen to account for both intercommissural (Al-Pm) distances at end diastole (37 mm) and at peak systole (35 mm) as well as the reduced annular dimension effect following quadrangular leaflet resection and annular plication. Virtual ring annuloplasty was performed by superimposing the intercommissural and septolateral lines of the ring and the annulus, and applying appropriate nodal displacements along the annulus to the ring configuration. Following the quadrangular resection and ring annuloplasty simulations, patient-specific annular motion and physiologic transvalvular pressure gradient were implemented and dynamic FE simulation of MV function was performed.

### Dynamic FE Simulation of MV Function over the Entire Cardiac Cycle

Patient-specific annular configurations at peak systole and end diastole were collected from the ECG-gated 3D TEE data, and dynamic annular motion was defined by time-varying nonlinear nodal displacement of the annulus between these two time points [23]. Annular displacement across the cardiac cycle was determined using the direction vectors from each nodal point along the annulus at peak systole and end diastole combined with previously reported ECG-gated annular motion data [23, 29].

Time-varying physiologic transvalvular pressure gradient over the entire cardiac cycle was applied to the ventricular side of the anterior and posterior leaflets. The maximum peak systolic and diastolic transvalvular pressure values were 15.4 kPa and -2.9 kPa [23]. The general contact algorithm was implemented to incorporate complete leaflet-to-leaflet and leaflet-to-chordae contact interactions [30]. The frictional coefficient was set to be 0.05 to simulate the physiologic frictional behaviors between the contact tissue during computational simulations of MV function [31].

## Results

### Echocardiographic Data vs. Computational Simulation

**Fig 3:**
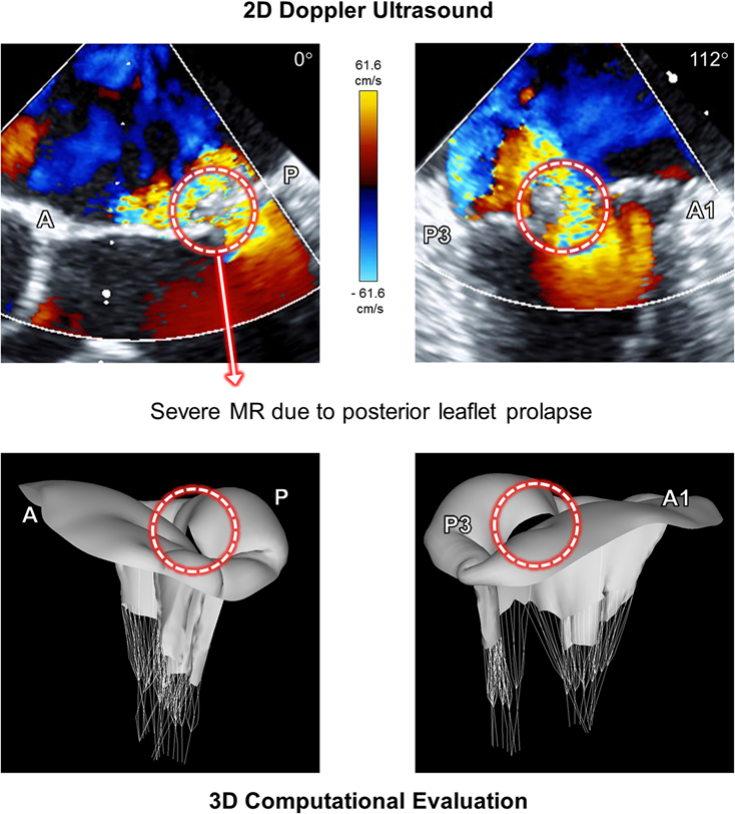
Doppler ultrasound images and the corresponding 3D morphologies of the MV of the pathologic MV involving posterior chordal rupture. Data shown at peak systole.

Fig 3 demonstrates the Doppler ultrasound images of the pathologic MV involving posterior chordal rupture in A-P and A1-P3 planes at peak systole, and the corresponding 3D morphologies of the MV obtained from the computational simulation. The 2D Doppler ultrasound data showed a large flail in the posterior leaflet and an anteriorly directed severe regurgitant jet at peak systole due to chordal rupture in the P2 scallop. Abnormal bulging of the posterior leaflet was clearly found in the 3D computational evaluation of MV function, which corresponds to the location and severity of MR in the Doppler ultrasound data. This indicates that our novel personalized computational modeling of MV prolapse provides valuable simulation outcomes to correctly represent the physiologic characteristics of patient-specific MV pathologies.

### Virtual Leaflet Resection and Ring Annuloplasty

**Fig 4:**
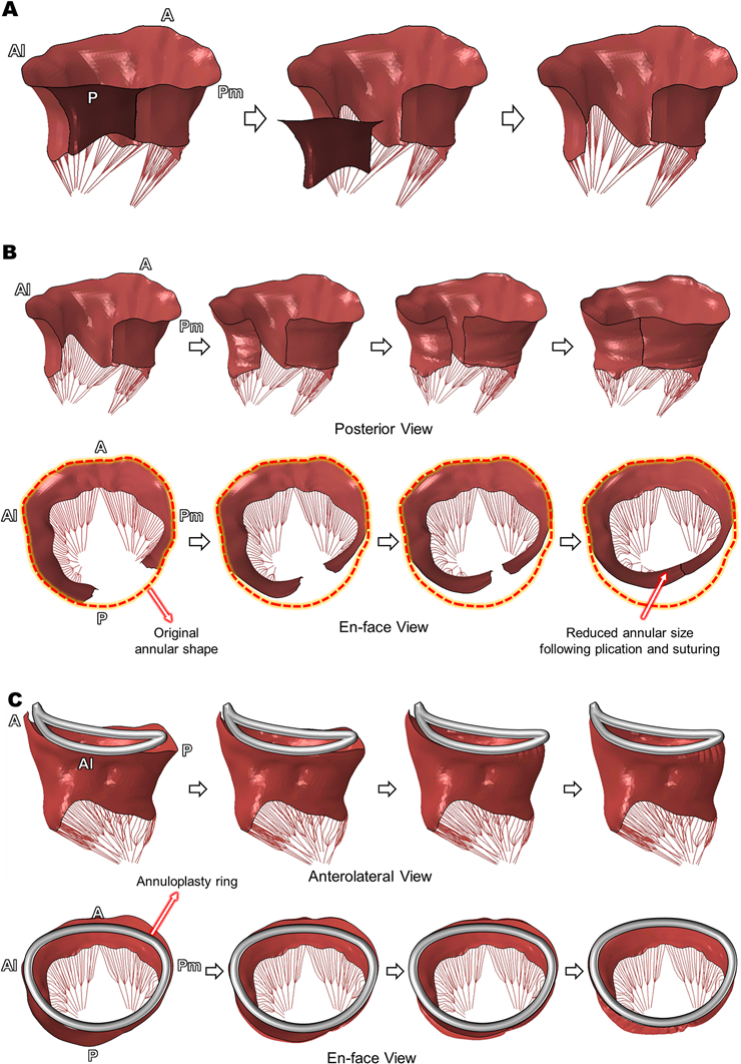
Virtual quadrangular posterior leaflet resection and ring annuloplasty. (A) Excision of the pre-defined leaflet tissue, (B) Virtual annular plication and suturing, (C) Virtual annuloplasty ring implantation.

Computational simulation of virtual quadrangular posterior leaflet resection and ring annuloplasty was successfully performed using the open configuration of the patient MV involving P2 chordal rupture (Fig 4). The pre-defined leaflet tissue for resection (dark red) was completely excised from the patient MV model (Fig 4A). Kinematic displacement of the excised leaflet edges effectively mimicked the subsequent annular plication and suturing procedures (Fig 4B). The posterior view provides information as to where the plication and suturing occurred, and the en-face view allows comparison of shape and size of the mitral annulus prior to and following the virtual quadrangular resection. The antero-posterior (A-P) and anterolateral-posteromedial (Al-Pm) annular diameters of the pre-resection MV involving posterior leaflet prolapse were 43 mm and 37 mm, respectively. The post-resection MV geometry revealed a 40% reduction in the A-P annular diameter (36 mm) while keeping the comparable Al-Pm distance (37 mm) during the plication and suturing procedures.

A Physio II ring of size 34 mm was modeled and virtually implanted [28] following the virtual resection procedure (Fig 4C). As the virtual quadrangular resection reduced the annular diameter in the P2-P3 region, the displacement of the annulus toward the ring configuration in the P1-P2 region was larger than other annular regions. The final dimension of the annulus fitted well to the 3D ring configuration and the annular nodal displacement was restricted to embody virtual suturing over the annuloplasty ring.

### Leaflet Stress Distribution Before and After Virtual MV Repair

**Fig 5:**
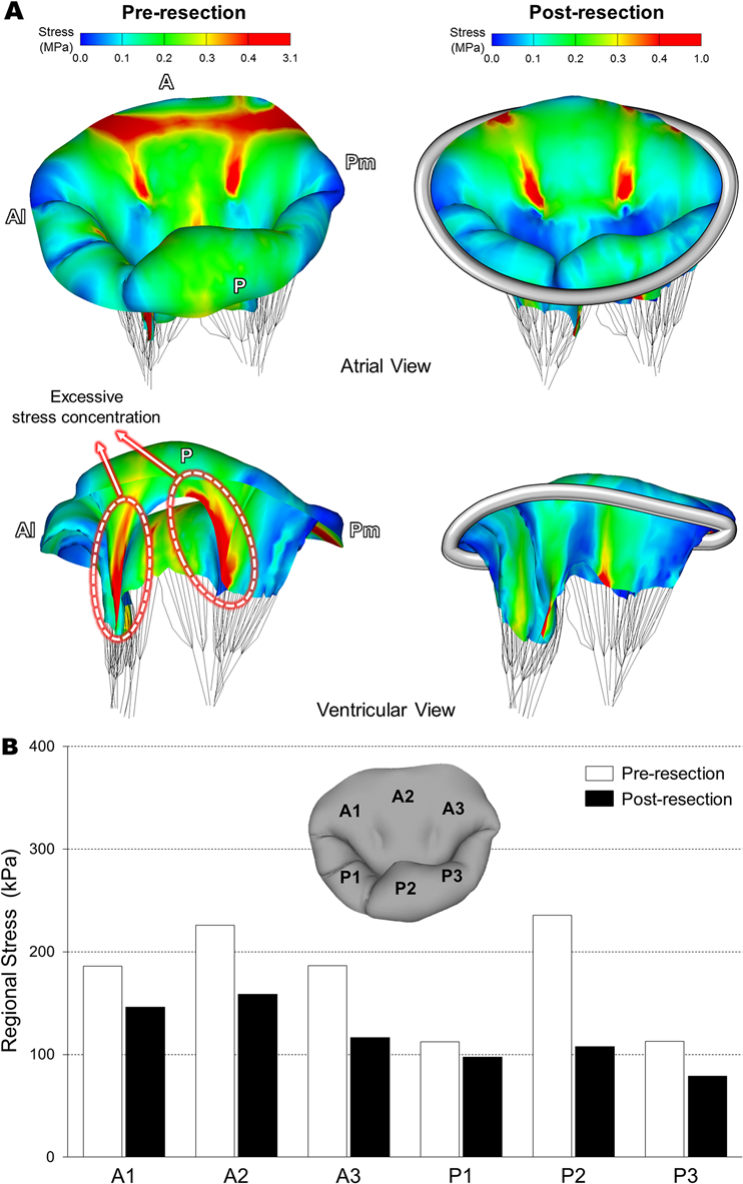
Stress distributions across the MV leaflets and annulus at peak systole. (A) Stress distributions prior to and following virtual posterior leaflet resection, (B) Average stress values in six sub-regions of the MV leaflets.

Fig 5 shows stress distributions across the MV leaflets and annulus at peak systole (i.e., fully closed position) prior to and following virtual posterior leaflet resection. To best demonstrate alterations of high stress concentration between the pre- and post-resection MVs, a threshold of stress value (0.4 MPa) was imposed on the simulation data such that stress values larger than this threshold were displayed in red.

Posterior leaflet prolapse was clearly observed in the pre-resection MV involving P2 chordal rupture (Fig 5A). High stress values occurred over a wide area near the mitral annulus-aorta junction spreading. Excessive stress concentration was found along the both sides of the free marginal edge of the posterior leaflet involving the chordal rupture (i.e., the P2 scallop). The maximum leaflet stress values (3.1 MPa) appeared near these P1-P2 and P2-P3 junction regions where the remaining intact posterior marginal chordae were connected. Following the virtual quadrangular posterior leaflet resection and ring annuloplasty, excessive stress concentration disappeared over both anterior and posterior leaflets. A large portion of the high stress concentration near the mitral annulus-aorta junction disappeared except the two locations where the strut chordae were connected. Reduction of the excessive stress concentration near the P1-P2 and P2-P3 junction regions was clearly found in the post-resection MV. There was no stress concentration around the annuloplasty ring. The maximum stress value (1.0 MPa) for the post-resection MV was much smaller than the pre-resection valve (3.1 MPa).

Average stress values in six sub-regions on the MV leaflets at peak systole are demonstrated in Fig 5B. As anticipated, the greatest decrease of regional stress following virtual MV repair was found in the P2 region. It is noteworthy that regional stresses in the entire anterior area (A1, A2, and A3) decreased more following virtual posterior leaflet resection compared to the P1 and P3 scallops. This indicates that posterior leaflet resection clearly affects stress distribution not only in the posterior leaflet, but also in the anterior leaflet due to restored leaflet coaptation.

### Leaflet Contact and Chordal Stress Distributions Before and After Virtual MV Repair

**Fig 6:**
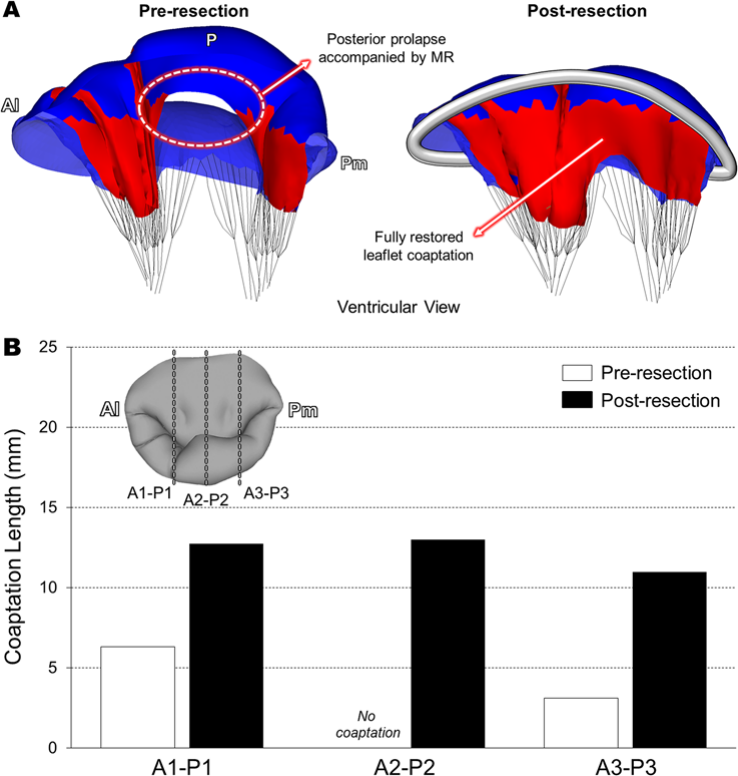
Quantitation of leaflet coaptation at peak systole. (A) Leaflet contact distribution prior to and following virtual MV repair, (B) Coaptation lengths in the A1-P1, A2-P2, and A3-P3 planes.

In order to determine the effect of virtual leaflet resection on restoration of coaptation, leaflet contact distribution and coaptation lengths at peak systole were assessed before and after the virtual MV repair (Fig 6). The pre-resection MV involving posterior chordal rupture demonstrated a substantial lack of leaflet coaptation over the prolapsed region which directly correlated with the severe MR (Fig 6A). Complete coaptation was found only in the vicinity of the anterolateral (A1/P1) and posteromedial (A3/P3) regions. Following the virtual posterior leaflet resection and ring implantation, the severity of the posterior leaflet prolapse markedly decreased and complete coaptation was effectively restored with sufficient contact between the anterior and posterior leaflets. Importantly, we found that the virtual MV repair improved leaflet coaptation not only in the P2 region where chordal rupture occurred, but also in the A1-P1 and A3-P3 regions (Fig 6B).

Reduced chordal stress distribution was clearly found in the post-resection MV (Table 1). Excessively large chordal stress values occurred in the C_4_ and C_11_ which are the two neighboring intact posterior chordae adjacent to the ruptured P2 scallop. Following virtual resection and ring annuloplasty, the average chordal stress decreased up to 62%.

**Table 1:** Average chordal stresses in the four neighboring intact posterior chordae at peak systole before and after virtual resection and ring annuloplasty.

		Pre-resection (kPa)	Post-resection (kPa)	Difference
P1/P2	C_3_	832	371	-56%
	C_4_	2,121	799	-62%
P2 (Ruptured)	C_5_–C_10_	N/A	N/A	N/A
P3	C_11_	2,953	1,315	-56%
	C_12_	254	208	-18%

## Discussion

Quadrangular resection combined with either an annular plication or a sliding leaflet approach are the most frequently performed surgical procedures to repair posterior leaflet prolapse [32–34]. Ring annuloplasty is the mainstay of all types of these repair procedure to correct an abnormally deformed mitral annulus and restore normal annular shape and size [2, 3]. Saddle-shaped annuloplasty rings provide the reconstruction of the nonplanar shape of the annulus possibly reducing leaflet stress [35]. If these surgical procedures for MV repair can be simulated virtually using computational techniques accompanied by patient-specific clinical imaging data, it is possible to perform personalized pre-surgical evaluation of a proposed MV repair intervention to assess and predict surgical outcomes. This novel strategy for virtual MV repair requires solid background and knowledge from both clinical and biomechanical perspectives. It is imperative to understand the physiology and pathology of the MV as well as to accurately perform personalized computational modeling of the MV pathology followed by virtual surgery using validated simulation methodologies. Computational MV evaluation can quantitatively assess the physiologic and biomechanical characteristics (e.g., detailed leaflet deformation, leaflet stresses, chordal stresses, leaflet coaptation, annular reaction forces, etc.) of MV function before and after MV repair. Current clinical echocardiographic techniques cannot provide this information. In the present study, we have developed a solid computational simulation protocol using standard clinical guidelines of posterior leaflet resection with annuloplasty ring implantation in a patient with posterior chordal rupture and severe MR.

Following the virtual posterior leaflet resection and ring annuloplasty, computational simulation of the post-repair MV function revealed markedly reduced stress concentration over both leaflets (Fig 5) and restored/enhanced leaflet coaptation across the intercommissural direction (Fig 6). It is likely that excessive leaflet stress concentration (up to 3.1 MPa) found along the both sides of the free marginal edge of the P2 scallop in the pre-resection MV may affect the remaining intact native chordae (Table 1) leading to additional chordal rupture or leaflet tissue failure. The post-resection MV showed a maximum stress value of 1.0 MPa, which is much lower than stress value of failing MV tissue [36]. This particular patient had complex pathophysiologic factors such as annular enlargement and posterior chordal rupture. Although these factors may involve complicated tissue characteristics in the pathologic MV, the personalized virtual MV repair simulation demonstrated good agreement with general clinical outcomes for improved leaflet coaptation with no regurgitation following leaflet resection and ring annuloplasty.

**Fig 7:**
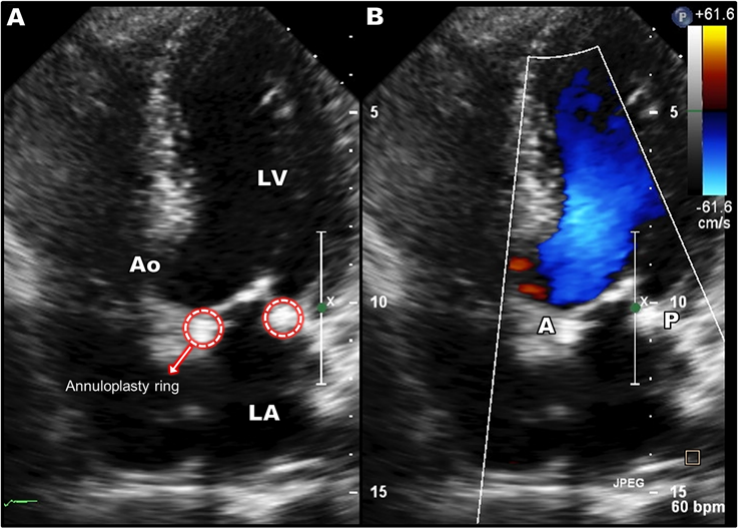
Post-repair Doppler ultrasound data following the actual surgical repair (quadrangular resection and ring annuloplasty) to treat the posterior MV prolapse.

The present virtual MV repair study was performed retrospectively. We collected the patient 3D TEE data and performed virtual posterior leaflet resection and ring implantation to restore normal MV function. The actual MV repair selected to treat the posterior leaflet prolapse of this patient was also quadrangular resection. Post-repair 2D Doppler ultrasound image is demonstrated in Fig 7. The operation successfully repaired the abnormal MV and restored normal valvular function. The selected annuloplasty ring size (34 mm) in the simulation was based on the standard clinical guidelines and the MV geometric information from the patient 3D TEE data taken prior to the operation (i.e., in vivo). There was good agreement to the actual ring size chosen by the surgeon (30 mm) for the operation when considering a slight reduction of the heart and annulus during bypass surgery. Our virtual MV repair strategy can be performed prospectively with results shared with surgeons preoperatively to help surgical planning.

There are several repair techniques utilized to treat posterior leaflet prolapse induced by chordal rupture. Another popular repair technique is neochordoplasty to reconstruct the ruptured chordae using artificial chordae. Studies have reported that neochordoplasty provides excellent preservation of leaflet mobility and larger surface coaptation [37, 38]. Both leaflet resection and neochordoplasty seem to be equally effective and comparable in short-, mid- and long-term clinical outcomes [39–41]. We have recently demonstrated personalized virtual neochordoplasty simulation techniques [18]. As there have been no studies reported to compare these two repair techniques from a biomechanical perspective, we are currently developing protocols to assess and compare the biomechanical effects of these two primary MV repair techniques on restoration of normal MV function.

In our computational MV modeling protocol, chordae insertion was distributed around the papillary muscle tips that were identified from 3D TEE data. The number of the chordae was selected from previous clinical studies [24, 42, 43] due to limited (both spatial and temporal) resolution of the currently available clinical 3D TEE data. In order to effectively model the posterior chordal rupture in this particular patient, the patient 3D TEE data were thoroughly examined and the partial absence of the chordae in the P2 region was appropriately modeled. This indicates that purely “personalized” computational MV modeling is not available yet with current 3D TEE imaging techniques. However, recent studies demonstrated the feasibility of visualization and accurate measurement of chordal lengths and distribution information from transgastric 3D TEE imaging [44]. Although our pre-resection MV simulation clearly demonstrated the physiologic characteristics of the patient MV involving posterior chordal rupture with severe MR (Figs 3, 5, and 6), it is anticipated that further advancement in clinical imaging modalities will improve computational modeling of the intact native chordae leading to more accurate and personalized simulation outcomes.

Another important modeling factor in our virtual resection protocol is the virtual suturing. We assumed that nodal displacements of the excised leaflet edges toward each other followed by combining the two edges can mirror the physical suturing during actual resection surgeries. There was no stress concentration nor abnormal deformation found around this virtually sutured location in the post-resection MV (Fig 5). It indicates that this computational strategy has successfully embodied the effect of physical suturing for the thin-layered human tissue (i.e., the MV leaflets).

The personalized computational modeling of MV prolapse utilizing patient 3D TEE data and virtual MV repair techniques presented in this study allows evaluation and prediction of physiologic valvular function before and after leaflet resection and ring annuloplasty. The clinical implications of this novel virtual MV repair strategy includes quantitative evaluation of MV pathologies, selection of surgical techniques, selection of the optimal shape and size of annuloplasty ring, determination of the extent of leaflet resection, and prediction of a desirable degree of post-operation leaflet coaptation, to obtain minimized MR, vanished physiologic abnormalities, and restored normal hemodynamics following MV repair. This novel personalized virtual MV repair strategy has great potential to help with preoperative selection of the patient-specific optimal MV repair techniques, allow innovative surgical planning to expect improved efficacy of MV repair with more predictable outcomes, and ultimately provide more effective medical care for the patient.
